# A longitudinal study on the relationships between maternal parenting style, children’s victimization, and self-esteem

**DOI:** 10.3389/fpsyt.2025.1573088

**Published:** 2025-10-28

**Authors:** Dandan Cheng, Cui Wang, Minghui Xiong, Ran Mo, Yuxuan Nie, Junsheng Liu

**Affiliations:** ^1^ College of Health Science, Shandong University of Traditional Chinese Medicine, Jinan, China; ^2^ Department of Psychology, School of Education, Soochow University, Suzhou, China; ^3^ College of Psychological and Cognitive Science, East China Normal University, Shanghai, China

**Keywords:** maternal warmth, maternal rejection, self-esteem, victimization, longitudinal study

## Abstract

**Background:**

Previous studies have established that parenting style and self-esteem affect children’s risk of victimization. This study further investigates the longitudinal mechanisms by which maternal warmth and rejection, along with self-esteem, influence children’s likelihood of victimization.

**Method:**

This study used a longitudinal design to examine the relationships between two key dimensions of maternal parenting styles (warmth and rejection), victimization, and self-esteem during middle and late childhood. It involved 607 third- and fourth-grade students and their mothers. A multi-informant approach assessed children’s victimization, self-esteem, and mothers’ parenting styles across three assessments over three years (T1, T2, and T3).

**Results:**

Self-esteem and maternal rejection negatively predicted each other, as did self-esteem and victimization at corresponding time points. By contrast, maternal warmth positively predicted self-esteem, and victimization was positively associated with maternal rejection. Additionally, victimization negatively predicted maternal warmth. The developmental cascade analysis demonstrated that self-esteem at T2 mediated the relationship between victimization at T1 and maternal rejection at T3. Similarly, self-esteem at T2 also mediated the relationship between maternal warmth at T1 and victimization at T3.

**Conclusions:**

These findings confirm the significant role of maternal parenting styles in fostering children’s self-esteem and reducing victimization, highlighting the dynamic interactions among key variables.

## 1 Introduction

Victimization refers to repeated, intentional harm inflicted by peers, from which individuals cannot adequately protect themselves (1). It is prevalent in primary and secondary schools (2) and is a significant risk factor for psychological difficulties in children, adolescents, and adults (3). Annually, approximately 246 million children and adolescents worldwide report experiencing abuse and violence at school (4). In China, about 20% of primary school children experience victimization (5). Victimized children are often marginalized, leading to significant psychological distress and serious risks to their physical and mental health. Specifically, victimization increases the likelihood of learning difficulties and exacerbates symptoms of anxiety and depression, potentially resulting in self-harm and suicidal behaviors (6, 7). Childhood victimization is a persistent form of harm that undermines overall health, with impacts extending into adulthood (8, 9). Thus, it is crucial to investigate factors contributing to victimization and identify strategies to reduce children’s exposure.

Among these factors, the family environment, particularly parenting style, strongly influences child victimization (10). The effects differ across styles. Negative parenting styles correlate with lower self-esteem in children, increasing their vulnerability to victimization (11, 12). Conversely, positive parenting styles serve as protective factors, reducing the risk of victimization (12, 13). This study examines two key dimensions, parental rejection and parental warmth, to understand how parenting style interacts with self-esteem and children’s victimization.

### 1.1 Parenting style and victimization

Many factors influence whether children are bullied, but parenting style is the most significant within the family system (14, 15). Parenting style encompasses parents’ attitudes, behaviors, and non-verbal communication, creating an emotional climate that affects the likelihood of children and adolescents being bullied (16–18). Baumrind’s two-dimensional model of parenting style, which includes responsiveness and demandingness, is highly influential (19–21). The combination of these dimensions yields four distinct parenting styles commonly identified in family research: authoritative, authoritarian, indulgent, and neglectful (21–23). Subsequent studies have further classified parenting styles, with parental warmth and rejection as key indicators of parenting behavior. Perris et al. developed the EMBU parenting questionnaire, comprising 15 subscales and 81 items (24). This tool assesses aspects such as over-involvement, affection, rejection, and parenting styles like overprotectiveness and guilt-inducing behavior. It has been widely adopted across various cultural contexts (25, 26).

Jiang et al. built on Perris et al.’s work and identified three parenting styles: rejection, emotional warmth, and excessive protection (27). Research indicates that warm, positive, democratic, and authoritative parenting enhances children’s self-concept, adaptive coping strategies in peer interactions, and social capacity for peer acceptance, thereby reducing the risk of victimization (28, 29). By contrast, overprotective parenting increases the likelihood of bullying victimization (30). Additionally, children who face parental rejection, neglect, or abuse, especially without close relationships or family support, are at a significantly higher risk of peer victimization (31). Parental acceptance-rejection theory suggests that individuals who feel rejected by their parents report more mental health and behavioral problems than those who feel accepted (32). Poor parental relationships, characterized by rejection or neglect, correlate with higher victimization rates among youth (33). Conversely, high parental involvement and support and a warm parent-child relationship are strong protective factors against harm for children and adolescents (34). Furthermore, children’s behavior can influence parenting practices; Hong et al. found a bidirectional relationship between strict parenting and children’s externalizing problems (35). This study investigates the interrelationships between maternal warmth, maternal rejection, and children’s victimization in the Chinese context.

### 1.2 Parenting style and children’s self-esteem

Parenting style significantly influences children’s self-esteem. Ecosystem theory posits that parental attention and care foster healthy self-evaluation and higher self-esteem, while denial and rejection diminish it (36). Numerous empirical studies support this relationship. For instance, Pinquart and Gerke conducted a meta-analysis of 116 studies, revealing that authoritative parenting correlates positively with self-esteem, whereas authoritarian and neglectful parenting are negatively associated (37). Children’s perceptions of parental care, support, involvement, and positive relationships enhance self-esteem (38). Conversely, perceived parental rejection, overprotection, and lack of emotional warmth can reduce self-esteem and predict the onset and persistence of externalizing behavior problems (39). Self-esteem can also affect parenting style; transaction theory suggests a reciprocal relationship between parenting and children’s functioning (40). Garaigordobil and Navarro found that high adolescent self-esteem buffers the negative impact of low parental acceptance. This protective effect suggests that a child’s strong self-esteem can mitigate the consequences of certain parenting styles, potentially encouraging more positive parental responses (41). In summary, positive parenting safeguards adolescents from victimization, whereas poor early parenting can hinder self-esteem development, increasing the risk of repeated victimization (42). This study examines two key parenting styles, parental warmth and parental rejection, and their impact on children’s self-esteem.

### 1.3 Children’s self-esteem and victimization

Self-esteem is an individual’s cognitive and emotional evaluation of their personal worth and ability (43). It develops through social interactions and is influenced by feedback from others. Self-esteem is multidimensional, developmental, and evaluative (44). Early victimization significantly predicts self-esteem levels: individuals with high self-esteem are at lower risk of being bullied, whereas those with low self-esteem face higher risks (45). Multiple forms of victimization can further reduce children’s self-esteem. According to social measurement theory, experiences of victimization may hinder young children’s social skills and instill self-doubt about their behavior and appearance, leading to negative self-evaluation and lower self-esteem (46). Low self-esteem depletes emotional and cognitive resources, making children and adolescents more likely to blame themselves for peer assaults, less capable of effective problem-solving, and more vulnerable to further victimization (47).

A meta-analysis by Mullan et al. indicated that bullied adolescents may experience lower self-esteem owing to negative peer evaluations that are internalized (48). Another study suggested that individuals with low self-esteem may be more susceptible to victimization as they seem unwilling or unable to defend themselves, increasing their risk of future victimization (49). As proposed by the transactional model, victimization and self-esteem are linked in a cyclical process, each reinforcing the other (45, 50). This study adopts a longitudinal design to investigate the dynamic reciprocal relationship between self-esteem and child victimization.

### 1.4 Parenting style, children’s self-esteem, and victimization

Positive parenting acts as a protective factor against school victimization (6, 34, 51). In Chinese culture, traditional gender roles—’male leads outside, female leads inside’—influence parenting behaviors. Mothers generally bear greater responsibility for nurturing and educating children and are more engaged in their daily lives than fathers. They serve as primary caregivers, providing comfort during distress (52). Roopnarine’s study of Afro-Caribbean immigrant families in the U.S. revealed a division of parenting roles, with mothers focusing on emotional and social development (53). Children exhibiting strong social skills and adaptability within the family are better positioned to establish positive peer relationships, reducing their victimization risk.

A mother’s warm and understanding parenting style promotes children’s self-esteem, whereas maternal punishment, harshness, and rejection can impede it. Research consistently shows a strong link between victimization and low self-esteem, often accompanied by depression and social isolation (54). Adolescents with low self-esteem are more susceptible to victimization because of their ineffective coping strategies in peer interactions, leading to increased social isolation and difficulty forming healthy relationships (55). Maternal warmth, characterized by emotional support and affection, meets children’s attachment needs and enhances psychological adjustment, allowing them to cope with distress and challenges more effectively (56). This process fosters resilience and stronger peer relationships, reducing the risk of victimization. Conversely, perceived maternal rejection can lead to negative self-perceptions and weakened self-esteem, resulting in socially withdrawn or submissive behaviors that elevate victimization risk (57, 58). This study examines mothers’ parenting styles in relation to children’s self-esteem and victimization.

### 1.5 The current study

This study employs a three-year longitudinal design based on the developmental cascade model. Four models were constructed: an autoregressive model (Model 1), a unidirectional model (Model 2), a bidirectional model (Model 3), and a full model (Model 4). These models systematically examine the reciprocal relationships between maternal parenting style, children’s experiences of victimization, and self-esteem in a Chinese context. The autoregressive model (Model 1) controls for variable stability over time, minimizing the effects of inertia on causal inference (e.g. the autoregressive path of maternal warmth from T1 to T3) (59), consistent with Darling and Steinberg’s assumption about parenting style stability (16). The unidirectional model (Model 2) emphasizes the dominant impact of parental behaviors in child development, such as the one-way prediction of self-esteem from warm parenting. The bidirectional model (Model 3) expands the traditional causal framework by incorporating feedback paths, such as the reciprocal relationship between self-esteem and victimization. This aligns with Sameroff’s transaction theory, which posits that individuals and their environments are mutually causal (50). For example, individuals with low self-esteem are more likely to face peer exclusion, which in turn diminishes their self-esteem, creating a negative cycle (49). The full model (Model 4) illustrates the complex network of cascading effects by integrating autoregressive, unidirectional, and bidirectional pathways. For instance, maternal warmth may indirectly lower the risk of victimization by enhancing self-esteem, whereas victimization experiences may diminish maternal emotional investment.

Previous studies have typically examined the mutual influence among these three variables using one-way prediction models, neglecting their dynamic interrelations (49). This study’s model architecture addresses this limitation by investigating the interaction pathways among parenting style (maternal warmth and maternal rejection), self-esteem, and victimization. We propose the following hypotheses:

1. Maternal warmth positively predicts children’s self-esteem, whereas maternal rejection negatively predicts it.
2. Children’s self-esteem negatively predicts their victimization.
3. Self-esteem mediates the relationship between maternal warmth (or maternal rejection) and children’s victimization. For example, maternal warmth at T1 influences children’s self-esteem at T2 and further influences the victimization of the children in T3.
4. There is a direct association between two variables, which operates in either a one-way or reciprocal manner.

## 2 Method

### 2.1 Participants

Grades 3 and 4 students from two primary schools in Jinan City and their mothers participated in the study. Data were collected across three sessions one year apart: December 2020 (T1), December 2021 (T2), and December 2022 (T3). A total of 653 questionnaires were collected at T1. Some participants were lost in subsequent sessions because of transfers, relocations, or other reasons. By the end of the three waves, 628 questionnaires were collected, yielding 607 valid responses after excluding missing or invalid data, representing a response rate of 96.66%. Among the respondents, 320 were boys (52.7%) and 287 were girls (47.3%). The mean age at T1 was 8.91 years (SD = 0.78).

At T1, there were 291 Grade 3 students (mean age = 8.35 years, SD = 0.55), comprising 153 boys (52.6%) and 138 girls (47.6%). In Grade 4, there were 316 students (mean age = 9.43 years, SD = 0.57), including 167 boys (52.8%) and 149 girls (47.2%). Maternal educational levels included 19.2% with junior high school or below, 34.9% with high school, 40.9% with basic or junior college, and 5.0% with a master’s degree or higher.

### 2.2 Measures

#### 2.2.1 Maternal parenting

Maternal parenting style was assessed using the Child-Rearing Practices Report, originally developed by Block and later revised by Chen et al. (60, 61). The questionnaire employs a 5-point Likert scale, prompting mothers to select the option that best reflects their parenting style. It includes dimensions such as acceptance, warmth, denial, rejection, and punishment. This study utilized five items to measure maternal warmth (e.g. ‘My child and I have warm, intimate times together’) and four items for maternal rejection (e.g. ‘I let my child know how ashamed and disappointed I am when s/he misbehaves.’), totaling nine items. Higher average scores indicate more frequent use of the respective parenting style. The questionnaire has been administered to Chinese samples, demonstrating good reliability and validity (62, 63). In this study, internal consistency coefficients for maternal warmth across three time points were 0.82, 0.80, and 0.84, while those for maternal rejection were 0.61, 0.67, and 0.67.

#### 2.2.2 Self-esteem

Self-esteem (e.g. ‘I like myself’) was assessed using the global self-worth subscale of the Self-Perception Profile for Children (64). This subscale includes six items rated on a five-point scale, with higher average scores indicating greater self-esteem. It has been widely used in China, demonstrating good reliability and validity (39, 63). In this study, internal consistency coefficients at three time points were 0.85, 0.89, and 0.93, respectively.

#### 2.2.3 Victimization

Peer victimization was assessed using six items from the revised Olweus Bully/Victim Questionnaire, adapted by Zhang et al. (65, 66). An example item is ‘I was called ugly nicknames, made fun of, and maliciously teased at school.’ Responses were scored on a 5-point scale, with higher average scores indicating greater victimization. The internal consistency coefficients across three time points were 0.86, 0.85, and 0.86, respectively.

### 2.3 Procedure

The study received approval from the internal review committee of the sponsoring university (Approval No.: HR2-0251-2021). Written consent was obtained annually from students and their parents prior to data collection. Group testing occurred at the class level, with assessments administered by senior undergraduates and postgraduates trained in psychology. Students completed paper-based questionnaires in class, which were collected immediately afterwards, whereas mothers completed their questionnaires online. Researchers provided instructions and assistance during classroom testing to facilitate comprehension and response. The link to the parenting style questionnaire was sent to mothers, and researchers maintained communication to support survey completion. Both mothers and students were assessed annually for three consecutive years using the same procedure.

### 2.4 Statistical analyzes

First, SPSS (version 25.0) managed missing values and generated descriptive statistics and correlation analyzes using multiple imputation. Second, Mplus 8.3 tested measurement invariance across the longitudinal data. Third, Mplus 8.3 constructed a series of nested models to examine developmental cascade effects among maternal parenting style, children’s self-esteem, and children’s victimization. A robust maximum likelihood estimator was used for model estimation. Maternal warmth and maternal rejection were separately entered into models, specifying four nested models for each: Model 1 (autoregressive), Model 2 (unidirectional), Model 3 (bidirectional), and Model 4 (full model). Chi-square difference tests compared these models to identify the optimal one that best captured the relationships among variables. Finally, the optimal model’s indirect effects were tested using the bias-corrected bootstrap method.

## 3 Results

### 3.1 Descriptive statistics and correlations

**Table 1** presents the means, standard deviations, and correlations among the three measures: maternal parenting style, self-esteem, and victimization.

**Table 1:** Descriptive statistics and correlational analysis.

Variable	*k*	*s*	*M SD*	1	2	3	4	5	6	7	8	9	10	11	12
1.T1 Maternal Warmth	25.62	18.24	4.23 0.65	1											
2.T1 Maternal Rejection	6.8	7.16	1.91 0.61	-0.25^***^	1										
3.T1 Child Self-Esteem	6.69	10.79	4.07 0.80	0.23^***^	-0.11^**^	1									
4.T1 Child Victimization	14.18	17.24	1.65 0.85	-0.21^***^	0.11^**^	-0.24^***^	1								
5.T2 Maternal Warmth	21.18	15.16	4.32 0.56	0.59^***^	-0.22^***^	0.13^**^	-0.13^**^	1							
6.T2 Maternal Rejection	10.56	8.35	1.91 0.61	-0.22^***^	0.48^***^	-0.14^**^	0.15^***^	-0.31^***^	1						
7.T2 Child Self-Esteem	4.14	10.81	3.94 0.93	0.45^***^	-0.03	0.42^***^	-0.21^***^	0.30^***^	-0.09^*^	1					
8.T2 Child Victimization	26.32	22.02	1.46 0.67	-0.13^**^	0.05	-0.17^***^	0.53^***^	-0.08^*^	0.12^**^	-0.22^***^	1				
9.T3 Maternal Warmth	20.88	17.59	4.25 0.74	0.37^***^	-0.22^***^	0.18^***^	-0.13^**^	0.36^***^	-0.25^***^	0.33^***^	-0.07	1			
10.T3 Maternal Rejection	15.42	11.79	1.86 0.63	-0.19^***^	0.46^***^	-0.04	0.06	-0.28^***^	0.49^***^	-0.11^**^	0.06	-0.31^***^	1		
11.T3 Child Self-Esteem	3.02	10.42	3.91 1.01	0.13^**^	-0.14^**^	0.24^***^	-0.13^**^	0.10^*^	-0.16^***^	0.33^***^	-0.11^**^	0.11^**^	-0.15^***^	1	
12.T3 Child Victimization	18.92	18.83	1.54 0.69	-0.17^***^	0.09^*^	-0.19^***^	0.39^***^	-0.11^**^	0.09^*^	-0.26^***^	0.50^***^	-0.18^***^	0.06	-0.30^***^	1

*p <.05, **p <.01, ***p <.001. The table indicates that maternal parenting style, self-esteem, and victimization were correlated across all three time points. Victimization at T1 negatively correlated with maternal warmth and self-esteem at T1, T2, and T3. Victimization at T2 showed negative correlations with maternal warmth at T1 and T2 and self-esteem at T1, T2, and T3. Similarly, victimization at T3 negatively correlated with both maternal warmth and self-esteem at all three time points. victimization at T1 positively correlated with maternal rejection at T1, T2, and T3, and victimization at T3 had significant positive correlations with maternal rejection at T1 and T2. Self-esteem at T1 negatively correlated with maternal rejection at T1 and T2; at T2, it negatively correlated with maternal rejection at T2 and T3; and at T3, it negatively correlated with maternal rejection at T1, T2, and T3.

### 3.2 Measurement invariance

**Table 2** presents the results of the measurement invariance analysis. The findings indicate that the measurement tool exhibited longitudinal invariance, confirming its effectiveness in assessing participants’ self-esteem, victimization, and parenting styles across different time points.

**Table 2:** Longitudinal measurement invariance.

Scale	χ^2^	c	*df*	CFI	RMSEA	SRMR	BIC	*Δ*CFI	*ΔRMSEA*
Self-esteem									
Configural MI	276.361	1.292	114	0.960	0.046	0.039	26472.11		
Weak MI	283.064	1.284	124	0.961	0.046	0.042	26414.26	-0.001	0.000
Strong MI	309.358	1.268	131	0.956	0.047	0.044	26398.18	0.005	-0.001
Victimization									
Configural MI	175.673	1.863	114	0.972	0.030	0.038	24127.53		
Weak MI	184.803	1.914	124	0.972	0.028	0.043	24089.89	0.000	0.002
Strong MI	211.303	1.854	132	0.964	0.031	0.043	24076.52	0.008	-0.003
Parenting									
Configural MI	957.589	1.148	294	0.803	0.063	0.091	29889.62		
Weak MI	959.317	1.167	310	0.807	0.061	0.093	29807.83	-0.004	-0.002
Strong MI	989.055	1.159	324	0.803	0.060	0.094	29745.93	0.004	0.001

### 3.3 Evaluation of the models

#### 3.3.1 Comparison of the models

To examine the relationships between maternal parenting style, children’s self-esteem, and victimization, a series of nested models was constructed: stability, unidirectional, bidirectional, and full models. Model fit was evaluated using indices: comparative fit index (CFI), Tucker–Lewis index (TLI), root mean square error of approximation (RMSEA), standardized root mean square residual (SRMR), and Akaike information criterion (AIC). A good model fit was defined as CFI and TLI values greater than 0.90, RMSEA and SRMR less than 0.08, and lower AIC values (67). Although chi-square significance tests were reported, they were not used as an absolute criterion for model fit (68).

The robust maximum likelihood estimator for non-normal data in Mplus was employed for path analysis, with the Satorra–Bentler χ2 difference test used to compare nested models. A significant chi-square difference test favored models with more complex paths; a non-significant test indicated a preference for more parsimonious structures (59). **Tables 3** and **4** present model fit indices and comparison results. Findings suggest that Model 4 consistently outperformed the other three models, whether examining maternal warmth or maternal rejection. Thus, Model 4 was selected for further investigation of the relationships between maternal parenting style, children’s self-esteem, and children’s victimization.

**Table 3:** Model fit indices for maternal warmth, children’s self-esteem, and victimization.

Model	*df*	c	*χ^2^*	CFI	TLI	RMSEA	SRMR	AIC	Model Comparison	cd	△*χ^2^*	*△df*
Model 1	18	1.18	132.69	0.85	0.73	0.10	0.09	11383.89				
Model 2	14	1.15	123.46	0.86	0.67	0.11	0.08	11377.91	1 *VS* 2	1.26	11.06^*^	4
Model 3	10	1.15	111.49	0.87	0.57	0.13	0.07	11372.42	2 *VS* 3	1.14	11.79^*^	4
Model 4	6	1.15	4.14	1.00	1.01	0.00	0.01	11256.68	3 *VS* 4	1.15	107.41^***^	4

Model 1 = autoregressive model; Model 2 = unidirectional model; Model 3 = bidirectional model; Model 4 = full model. Applies to subsequent tables.

*p < .05, ***p < .001.

**Table 4:** Model fit indices for maternal rejection, children’s self-esteem, and victimization.

Model	df	c	χ2	CFI	TLI	RMSEA	SRMR	AIC	Model Comparison	cd	△χ2	△df
Model 1	18	1.08	62.30	0.94	0.89	0.06	0.06	11239.38				
Model 2	14	1.09	52.15	0.95	0.88	0.07	0.05	11236.95	1 *VS* 2	1.04	10.01^*^	4
Model 3	10	1.08	28.30	0.98	0.92	0.06	0.03	11218.84	2 *VS* 3	1.11	23.63^***^	4
Model 4	6	1.14	10.36	0.99	0.97	0.04	0.02	11208.12	3 *VS* 4	0.99	18.93^***^	4

* *p*< .05, *** *p*< .001.

#### 3.3.2 Developmental cascade effects of maternal warmth, self-esteem, and victimization

**Figure 1:**
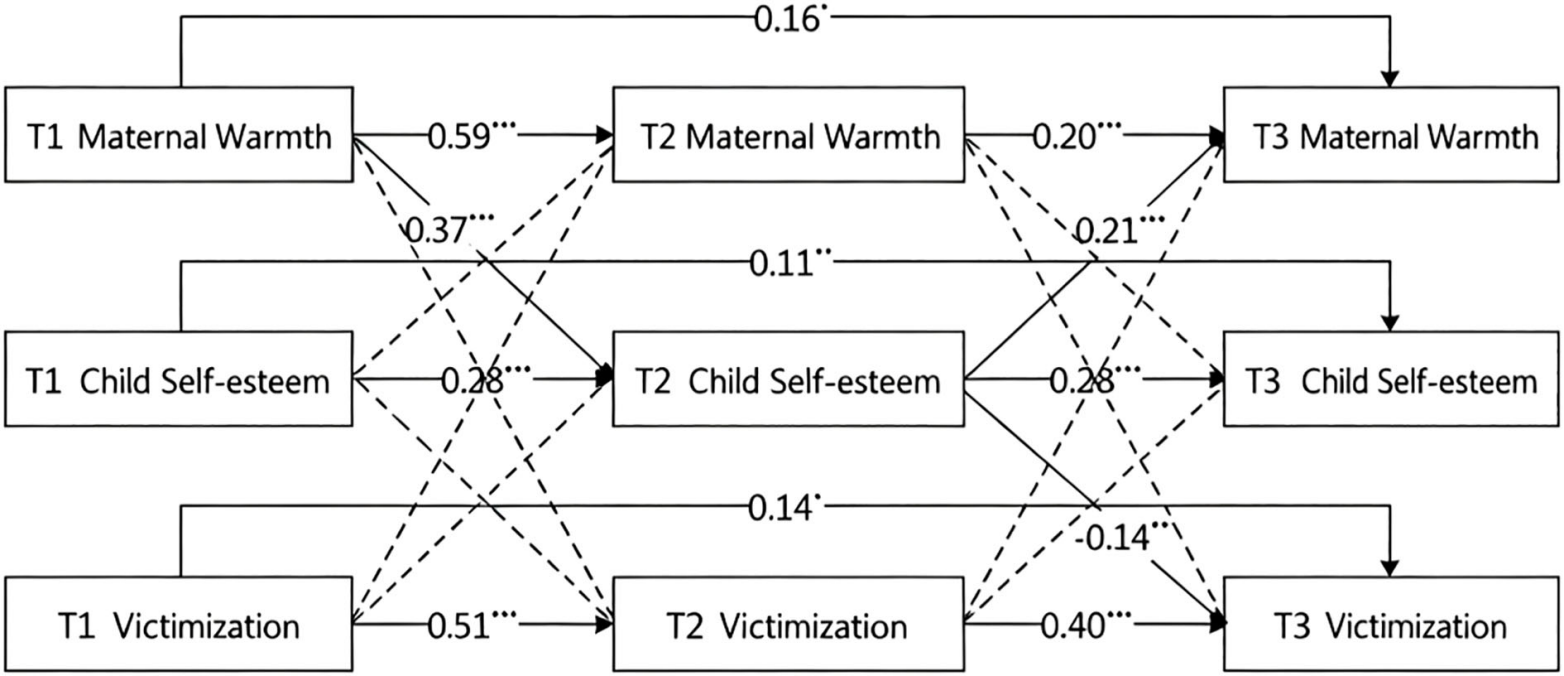
Cascade model of maternal warmth. ^*^ *p* <.05; ^**^ *p* <.01; ^***^ *p* <.001.

As shown in **Figure 1**, all autoregressive pathways in the maternal warmth model were significant, indicating stability among maternal warmth, children’s self-esteem, and victimization. After controlling for variable stability and correlations at the same point, maternal warmth at T1 significantly predicted children’s self-esteem at T2 (*β* = 0.37, *p* <.001). In turn, children’s self-esteem at T2 significantly predicted maternal warmth at T3 (*β* = 0.21, *p* <.001) and children’s victimization at T3 (*β* = -0.14, *p* <.001).

To examine potential indirect effects, a bias-corrected bootstrap method with 5,000 samples was used. The standardized coefficients and 95% bias-corrected confidence intervals for all significant indirect effects are presented in **Table 5**. Four indirect pathways were identified: (i) T2 self-esteem mediated the association between T1 maternal warmth and T3 self-esteem; (ii) T2 self-esteem mediated the association between T1 maternal warmth and T3 victimization; (iii) T2 self-esteem mediated the association between T1 self-esteem and T3 victimization; and (iv) T2 self-esteem mediated the association between T1 self-esteem and T3 maternal warmth. In summary, T2 self-esteem served as a mediator linking T1 maternal warmth to T3 victimization, T3 self-esteem, and T3 maternal warmth, indicating mutual influence among these variables.

**Table 5:** Indirect path significance test for cascade model of maternal warmth.

Mediation paths	Effect	SE	95% CI
Maternal Warmth(T1)→Self-Esteem(T2)→Self-Esteem(T3)	0.10^***^	0.02	(0.06, 0.15)
Maternal Warmth(T1)→Self-Esteem(T2)→Victimization(T3)	-0.05^**^	0.02	(-0.09, -0.02)
Self-Esteem(T1)→Self-Esteem(T2)→Victimization(T3)	-0.05^**^	0.02	(-0.08, -0.02)
Self-Esteem(T1)→Self-Esteem(T2)→Maternal Warmth(T3)	0.07^***^	0.02	(0.03, 0.10)

*p < .05, ***p < .001.

#### 3.3.3 Developmental cascade effects of maternal rejection, self-esteem, and victimization

**Figure 2:**
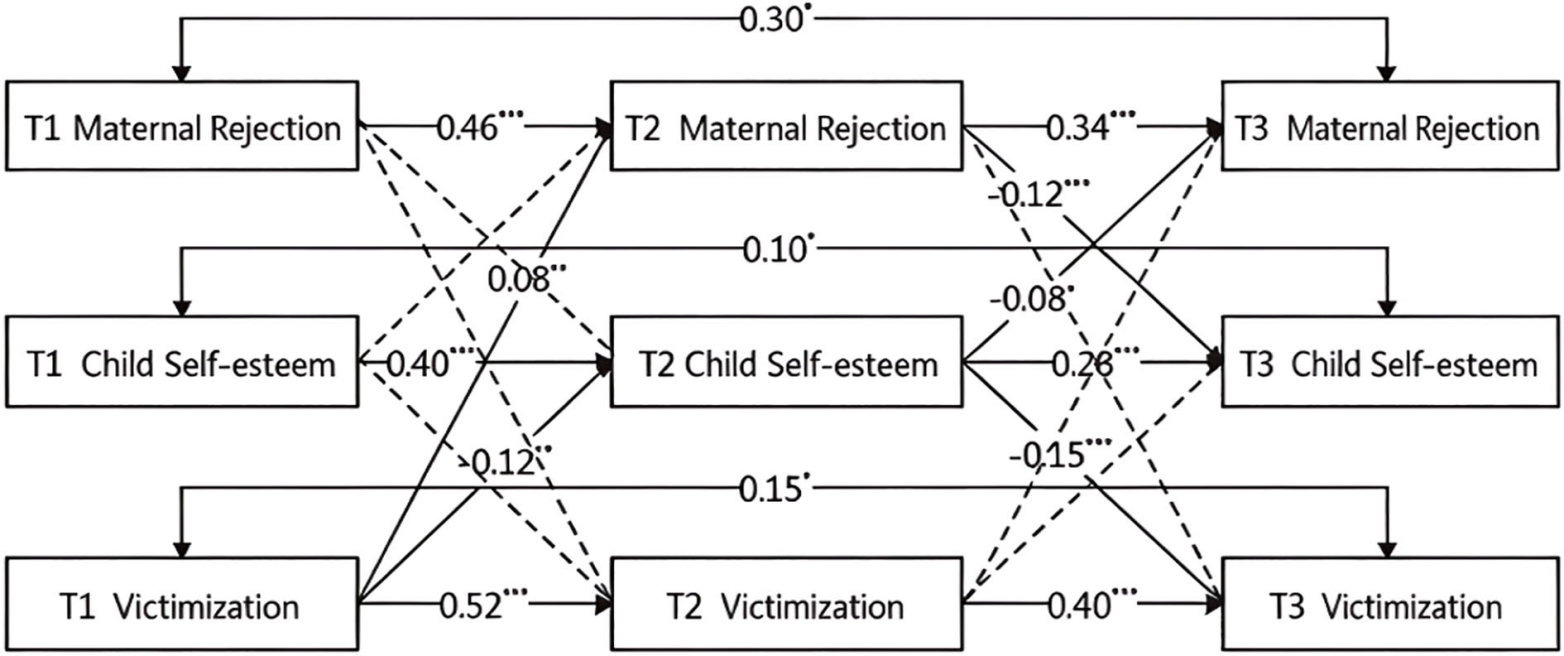
The cascade model of maternal rejection. *p <.05; **p <.01; ***p <.001.

As shown in **Figure 2**, all autoregressive paths in the maternal rejection model were significant, indicating stability among maternal rejection, children’s self-esteem, and victimization. After controlling for variable stability and correlations at the same time point, children’s victimization at T1 significantly predicted maternal rejection at T2 (*β* = 0.08, *p* <.01) and children’s self-esteem at T2 (*β* = −0.12, *p* <.01). Maternal rejection at T2 significantly predicted children’s self-esteem at T3 (*β* = −0.12, *p* <.001). Additionally, children’s self-esteem at T2 significantly predicted maternal rejection at T3 (*β* = −0.08, *p* <.05) and children’s victimization at T3 (*β* = −0.15, *p* <.001).

To further examine possible indirect effects, a bias-corrected bootstrap method with 5,000 samples was applied based on the direct effects. The standardized coefficients and 95% bias-corrected confidence intervals for all statistically significant indirect effects are reported in **Table 6**. Five indirect pathways were identified: (i) T2 maternal rejection mediated the association between T1 maternal rejection and T3 self-esteem; (ii) T2 self-esteem mediated the association between T1 victimization and T3 self-esteem; (iii) T2 self-esteem mediated the association between T1 self-esteem and T3 victimization; (iv) T2 maternal rejection mediated the association between T1 victimization and T3 maternal rejection; and (v) T2 self-esteem mediated the association between T1 self-esteem and T3 maternal rejection. In summary, T2 self-esteem mediated the links from T1 victimization to T3 self-esteem, T3 victimization, and T3 maternal rejection, indicating reciprocal influence among the three variables.

**Table 6:** Indirect path significance test table for cascade model of maternal rejection.

Mediation paths	Effect	SE	95% CI
Maternal Rejection(T1)→Maternal Rejection(T2)→Self-Esteem(T3)	-0.06^**^	0.02	(-0.09, -0.02)
Victimization(T1)→Self-Esteem(T2)→Self-Esteem(T3)	-0.03^*^	0.01	(-0.06, -0.01)
Self-Esteem(T1)→Self-Esteem(T2)→Victimization(T3)	-0.06^***^	0.02	(-0.09, -0.02)
Victimization(T1)→Maternal Rejection(T2)→Maternal Rejection(T3)	0.03^*^	0.01	(0.01, 0.05)
Self-Esteem(T1)→Self-Esteem(T2)→Maternal Rejection(T3)	-0.03^*^	0.01	(-0.06, -0.00)

*p < .05, **p < .01, ***p < .001.

## 4 Discussion

This study examined the relationships between maternal parenting style, children’s self-esteem, and children’s victimization over a three-year follow-up. Maternal warmth, maternal rejection, children’s self-esteem, and children’s victimization showed no significant growth during this period. The associations between maternal parenting style and victimization, as well as between self-esteem and victimization, were consistent with our hypotheses, including the mediating effects of self-esteem. However, maternal parenting style and victimization did not influence each other longitudinally, contradicting our hypothesis and the findings of Lereya et al. (34). A possible explanation is that the sample may have changed owing to attrition and shifts in participant characteristics. By contrast, cross-sectional studies use a fixed sample at one point in time. Additionally, different measurement tools and methods in longitudinal and cross-sectional studies may introduce systematic errors that affect the accuracy of the results. This study applied the developmental cascade model to control for variables over time and investigate dynamic changes, which may explain inconsistencies in findings. These results have practical implications for parents, suggesting ways to reduce children’s victimization, lower its frequency, promote healthier development through adaptive parenting styles, and strengthen children’s self-esteem.

### 4.1 The relationship between maternal parenting style and self-esteem

Maternal parenting style significantly predicted self-esteem, demonstrating a dynamic bidirectional relationship, thereby supporting Hypotheses 1 and 4. Maternal warmth at T1 positively predicted self-esteem at T2, and self-esteem at T2 positively predicted maternal warmth at T3. Maternal rejection at T2 negatively predicted self-esteem at T3, and self-esteem at T2 negatively predicted maternal rejection at T3. These findings align with previous studies, indicating that children raised in positive parenting environments—characterized by warmth and understanding—receive encouragement from their parents, leading to improved self-esteem (38). Conversely, negative parenting styles, such as rejection or overprotection, diminish children’s autonomy and enthusiasm, potentially resulting in frustration and lower self-confidence (39). Furthermore, individuals with low self-esteem may externalize failures as aggression (69), reducing the warmth and understanding they receive from parents, thereby straining the parent-child relationship. This strain may lead parents to adopt more rejecting styles in their interactions (70). These results resonate with findings from both Chinese and European families, showing that authoritarian parenting harms children’s self-esteem and development, whereas parental care fosters positive growth (71, 72). A key factor is how loved, valued, and appreciated children feel within the family (73). Consistent with this study and recent research on Chinese families, children’s self-esteem increases within close, engaged, and caring relationships (71, 74, 84).

### 4.2 The relationship between self-esteem and victimization

Self-esteem significantly predicted children’s victimization, supporting Hypothesis 2. Regardless of maternal warmth or rejection, self-esteem at T2 significantly affected victimization at T3. Additionally, within the context of maternal rejection, victimization at T1 negatively predicted self-esteem at T2, supporting Hypothesis 4. This indicates a reciprocal influence between self-esteem and victimization under maternal rejection, consistent with findings by Eijnden et al. (75). Self-esteem is shaped by social acceptance and external evaluations, with both objective experiences and subjective perceptions of rejection having detrimental effects on self-esteem (76). Bullied children face rejection and unfair treatment from peers, undermining their self-evaluation and emotional well-being, which threatens their self-esteem (49). Adolescents with low self-esteem are often perceived as easy targets, increasing their risk of victimization. This may stem from their cautious behavior and poor self-regulation when bullied, limiting their ability to defend themselves and increasing the likelihood of repeated victimization (75). The frequency and intensity of victimization correlate with lower self-worth, self-attitude, self-satisfaction, and overall self-esteem. Individuals with low self-esteem tend to interpret external information negatively (77), perceiving others as more aggressive or hostile, which deteriorates peer relationships and further heightens the risk of victimization.

### 4.3 The mediating role of self-esteem

Children’s self-esteem mediated the influence of maternal warmth on victimization and the impact of victimization on maternal rejection, supporting Hypothesis 3. Specifically, maternal warmth at T1 affected victimization at T3 through self-esteem at T2, whereas victimization at T1 affected maternal rejection at T3 through self-esteem at T2. A warm parenting style fosters children’s feelings of care and support, facilitating the development of self-esteem and self-confidence. Higher self-esteem correlates with stronger self-confidence, leading to positive interpersonal relationships and a reduced likelihood of victimization. Frequently bullied individuals often experience depression and anxiety (6), lowering their self-perception. Children with low self-esteem seldom communicate with parents about school experiences, making it difficult to seek support when bullied. A cold or indifferent family atmosphere can exacerbate rejecting parenting styles (78). This finding indicates that self-esteem mediates the relationship between parenting style and children’s victimization.

### 4.4 The mediating role of maternal rejection

This study found that, in addition to self-esteem, maternal rejection mediates a specific pathway: victimization at T1 affects self-esteem at T3 through maternal rejection at T2. According to the parental acceptance–rejection theory, parenting styles can shape children’s victimization experiences (79). However, results indicated that parenting practices did not directly predict later victimization. Instead, child victimization predicted increased maternal rejection at the subsequent time point. One possible explanation is that victimization may lead to negative behaviors, such as social withdrawal, which parents perceive as sources of stress, triggering rejecting parenting styles (80–82). Victimization at T2 predicted negative parenting behaviors at T3. Consistent with this, studies show that children who are bullied often perceive greater parental rejection (72). When confronted with their child’s victimization, parents may experience psychological pressure from self-blame or heightened anxiety, leading to withdrawal of emotional support and more rejecting parenting styles (73). Additionally, parents who do not understand their children’s emotional needs may attribute victimization to weakness or perceive it as normal, responding with indifference (83). This intensifies rejection behaviors. Children rejected by their mothers may engage in self-denial, lowering their self-esteem. In conclusion, this study contributes to evidence that children’s behaviors can influence parental behavior. Maternal rejection mediates the relationship between victimization and self-esteem; however, no comparable pathway was identified for maternal warmth, indicating a need for further research.

## 5 Strengths and limitations

This study has several strengths. First, it utilized a large longitudinal dataset to examine changes in maternal parenting style, children’s self-esteem, and victimization over time. By following participants of varying ages, the study captured the dynamics among these variables, illustrating how maternal parenting style influences children’s self-esteem, which in turn affects their victimization status. This longitudinal method is more effective than a cross-sectional design, which only observes participants at a single time point and fails to capture developmental changes.

Second, the study employed a three-wave longitudinal design to investigate the distinct maternal warmth and rejection mechanisms. It revealed the bidirectional interactions among parenting styles, self-esteem, and victimization. The findings confirmed that self-esteem mediates the relationship between maternal warmth (and rejection) and children’s victimization, while also showing that victimization experiences can lead to increased maternal rejection. This dynamic reveals a vicious cycle: victimized children may provoke negative responses from mothers, exacerbating their psychological vulnerability. Consequently, the study enhances our understanding of parent-child interactions by emphasizing the reciprocal process of ‘two-way shaping’ between parents and children.

This study produced significant results but has several limitations. First, it relied on participants’ self-reports, which, although valuable for capturing victims’ experiences, are susceptible to cognitive biases and memory distortions. These can create discrepancies between subjective reports and actual events. Future research should enhance data reliability by incorporating experimental designs and in-depth interviews. Second, using observed variables rather than latent variables limited the depth of analysis. Future studies should identify latent variables from multiple indicators and examine the impact of parenting styles on children individually and collectively across various dimensions, providing more targeted guidance for caregivers. Finally, the sample was drawn from a specific cultural context, limiting the generalizability of the findings. To improve generalizability, future research should include diverse age groups and participants from various regions to assess the cross-group and cross-cultural applicability of the proposed model.

## 6 Conclusions and implications

This study employed three waves of longitudinal data to examine the relationships among parenting style, self-esteem, and victimization. Findings indicated that maternal warmth at T1 influenced self-esteem at T2, which predicted victimization at T3, suggesting a mediating role for self-esteem. A dynamic interaction between maternal warmth and self-esteem was observed over time. By contrast, a bidirectional relationship was identified between victimization and self-esteem within the maternal rejection dimension, with maternal rejection at T2 mediating the association between victimization at T1 and self-esteem at T3. Overall, the relationships among parenting style, self-esteem, and victimization are characterized by a dynamic process over time. These findings enhance scholarly understanding and extend the theoretical framework of dynamic mechanisms in victimization research. Practically, the study underscores the need for targeted interventions that include (i) guiding mothers to strengthen positive parenting behaviors, such as maternal warmth, to enhance children’s self-esteem, and (ii) the timely identification and support of children showing signs of peer victimization, which can disrupt the negative cycle leading to further maternal rejection. Such strategies can help break the feedback loop in mother–child interactions and reduce the risk of long-term abuse.

## Data Availability

The raw data supporting the conclusions of this article will be made available by the authors, without undue reservation.
